# Childhood sepsis burden: pathogens/antimicrobial-resistant bacteria, 1990–2021 and 2050 forecasts

**DOI:** 10.3389/fcimb.2026.1855912

**Published:** 2026-06-19

**Authors:** Weimin Zhu, Pinlu Jiang, Jiansong Yu, Tianyu Shen, Xule Yang, Yinghe Xu, Shengwei Jin, Yongpo Jiang

**Affiliations:** 1 Department of Critical Care Medicine, Taizhou Hospital of Zhejiang Province Affiliated to Wenzhou Medical University, Taizhou, Zhejiang, China; 2 Department of Emergency Medicine, Taizhou Hospital of Zhejiang Province affiliated to Wenzhou Medical University, Taizhou, Zhejiang, China; 3 Zhejiang Engineering Research Center for Intelligent Medical ImagingSensing and Non-invasive Rapid Testing, Taizhou, Zhejiang, China; 4 Department of Anesthesia and Critical Care, The Second Affiliated Hospital and Yuying Children’s Hospital of Wenzhou Medical University, Wenzhou, Zhejiang, China

**Keywords:** antimicrobial resistance, childhood sepsis, global burden of disease, pathogens, public health intervention

## Abstract

**Background:**

To assess the global burden and trends of childhood sepsis, and to evaluate whether clarifying these aspects can provide a basis for formulating targeted prevention and treatment strategies for the condition.

**Methods:**

Using the Global Burden of Disease (GBD) 2021 database, we analyzed sepsis in 0–14-year-olds, covering 62 pathogens (21 drug-resistant), 84 “pathogen–drug” combinations, 12 infection syndromes, and indicators (deaths, disability-adjusted life years [DALYs], age-standardized rates). Antimicrobial resistance (AMR) was defined by two counterfactual scenarios; an autoregressive integrated moving average (ARIMA) model forecast AMR-related sepsis to 2050.

**Results:**

From 1990 to 2021, global sepsis-related deaths among children declined from 6.43 million to 2.24 million, and DALYs dropped from 578 million to 203 million. The estimated annual percentage change (EAPC) in age-standardized mortality rate (ASMR) and DALY rate (ASDR) was –3.35 and –3.33, respectively. AMR-related sepsis burden also decreased, with notable declines in both AMR-associated and AMR-attributable deaths and DALYs. In 2021, the highest burden remained in Sub-Saharan Africa, while the lowest was in High-income regions. Leading pathogens causing child sepsis deaths included Streptococcus pneumoniae, Klebsiella pneumoniae, Escherichia coli, Staphylococcus aureus, and Pseudomonas aeruginosa. Bloodstream infections, lower respiratory infections, and diarrhea were the most common infection syndromes leading to sepsis-related deaths.

**Conclusion:**

Global childhood sepsis burden dropped, but high-burden regions (e.g., Sub-Saharan Africa, South Asia) need focus. Future efforts should optimize healthcare, strengthen AMR control, promote vaccination, and improve sanitation.

## Introduction

Sepsis, which involves life-threatening organ dysfunction caused by an improper immune response to infection, presents a serious danger to the health of children, especially newborns, who often face a higher risk of infection due to factors such as prematurity (Schlapbach et al., 2024). According to the GBD 2017 study, there were approximately 20 million cases of sepsis among children under 5 years of age worldwide, and an additional 5 million cases among children aged 5 to 19, resulting in 3.5 million deaths, children account for half of all sepsis cases and one-third of sepsis-related deaths globally (Rudd et al., 2020). The disease burden is particularly severe in Africa, South America, and the Indian subcontinent (Menon et al., 2022). In some resource-limited settings, the case-fatality rate of pediatric sepsis can be as high as 25% to 50% (Tan et al., 2019).

Despite significant progress in child health globally and the declining incidence of traditional pathogens such as Streptococcus pneumoniae and Hemophilus influenzae due to widespread vaccination (Group PERfCHPS, 2019), the misuse and overuse of antimicrobial agents have become increasingly prevalent, accelerating the emergence and spread of AMR and further threatening children’s health (Huemer et al., 2020; Collaborators AR, 2022). AMR has become a significant worldwide public health issue. If appropriate measures are not taken to address this issue, the number of deaths caused by AMR infections is expected to continue rising in the coming years (Pulingam et al., 2022; Collaborators GAR, 2024). The Lancet published a comprehensive global study indicating that in 2019, AMR was connected to nearly 5 million deaths, with 1.4 million in South Asia and 1 million in Sub-Saharan Africa., among the 5 million sepsis-related deaths linked to AMR, over 1 million involved children under the age of 5 (Collaborators AR, 2022). Children in resource-limited settings with limited access to health services face a heightened risk; the lack of safe water, poor sanitation, inadequate hygiene practices, and weak infection control measures greatly accelerate the spread of AMR, and if bacteria develop resistance to key therapeutic agents like antiretroviral drugs, antimalarials, antituberculosis drugs, and antifungals, the progress made over decades in reducing child mortality may be significantly undermined (WHO, 2020a).

However, current research on the burden of sepsis caused by bacterial pathogens and AMR is predominantly focused on adults, with relatively few systematic studies dedicated specifically to children (Collaborators GAR, 2022). The immune system of children is not yet fully developed, and they differ from adults in terms of the types and routes of bacterial infections, clinical manifestations, and responses to antimicrobial treatments. Moreover, children’s living environments, sanitation conditions, and vaccination coverage vary widely across regions, all of which influence the epidemiology and resistance patterns of bacterial pathogens. Therefore, a comprehensive and systematic analysis of the changing trends in the global burden of sepsis caused by bacterial pathogens and AMR among children from 1990 to 2021, along with projections up to 2050, is essential for developing focused prevention and control tactics to advance child health. This study aims to address some of the existing research gaps and provide strong scientific evidence to support global efforts in promoting child health.

## Methods

### Data sources

The GBD 2021 adopted the latest epidemiological data and optimized standardized methodologies to comprehensively assess health losses attributable to 371 diseases, injuries and impairments, as well as 87 risk factors across 204 countries and territories by age and sex (Collaborators GDaI, 2020). It integrates diverse data sources with exclusive identification codes, all of which are archived in the Global Health Data Exchange (GHDx). To estimate the global burden of childhood sepsis caused by pathogenic bacteria and AMR from 1990 to 2021, relevant data were extracted from the GBD 2021 dataset. This dataset includes the disease burden attributable to 62 pathogens (including 21 drug-resistant pathogens), 84 “pathogen–drug” combinations, and 12 infection syndromes during the period 1990–2021. Detailed methodologies have been published elsewhere (Collaborators GAR, 2022; Collaborators GAR, 2024). All data used in this study were sourced from publicly available databases, and therefore, ethical review and informed consent were not required.

### Data analysis

This study described the disease burden of sepsis caused by pathogens and AMR among children aged 0–14 years globally in 1990 and 2021, including the number of deaths, DALYs, and their corresponding age-standardized rates (ASR). All estimates were reported with 95% uncertainty intervals (UI), which incorporated uncertainties from data sampling, adjustments for differences in data sources for all-cause mortality, and model specifications for cause-of-death estimation. The UI was generated by taking 1000 draws from the posterior distribution of each quantified indicator. To assess the overall trend in the burden of childhood sepsis, we used the EAPC. When comparing populations with different age structures or analyzing shifts in age composition over time, standardization is vital, the ASR trends analyzed through EAPC were considered more reliable indicators for tracking changes in disease patterns (Liu et al., 2019). The direct age-standardized rate was calculated using the following formula:


$$ASR=\frac{\sum_{\text{i}=1}^{A}a_{i}w_{i}}{\sum_{\text{i}=1}^{A}w_{i}}\times 10,000$$

where ai and wi represent the specific age rates and the number of individuals in the corresponding age group of the selected reference population (or weights), where i indicates the i-th age group. A linear regression model was constructed in the form of *y = α + βx*, where *y* represents the natural logarithm of ASR (ln [ASR]), and *x* represents the calendar year. The EAPC was calculated as (exp[*β*] − 1) × 100%, with the 95% confidence interval (CI) derived from the regression model.

### Attributable to drug-resistance and associated with drug-resistant infections

The methods for calculating the impact of AMR have been outlined before (Collaborators GAR, 2024; Zhang et al., 2024). We conducted a systematic review of the burden of infectious diseases among children aged 0–14 years in 2021, covering 12 infection syndromes and 62 pathogens. The burden of AMR was assessed for 21 drug-resistant pathogens and 84 combinations of pathogens and drugs. Considering the uncertainty about whether resistant infections would vanish or be replaced by susceptible ones without AMR, we evaluated the AMR burden in two alternative scenarios: one presuming no infections (i.e., burden associated with AMR), and the other presuming replacement by infections (i.e., burden attributable to AMR). The AMR-attributable burden was quantified as the product of five metrics: the number of deaths attributable to drug-resistant infections, the proportion of these deaths complicated by sepsis, the proportion of sepsis deaths attributable to specific infectious syndromes, the proportion of syndrome-specific deaths attributed to individual pathogens, and the mortality population attributable fraction (PAF) for each resistance phenotype. The analytical framework for AMR-associated burden was identical to that for AMR-attributable burden, with the mortality PAF replaced by the resistance prevalence among all deaths for each resistance profile. Using the standard GBD approach (Collaborators GDaI, 2024), age-specific deaths were converted into years of life lost (YLLs) based on age-specific counterfactual life expectancy. Years lived with disability (YLDs) were calculated by multiplying the incidence of infectious syndromes, the pathogen-attributable proportion of syndrome cases, the per-case YLD estimate, and the non-fatal PAF. DALYs were calculated as the sum of YLLs and YLDs. For pathogens with multi-class antibiotic resistance, the total AMR burden was proportionally redistributed to individual antibiotic classes according to excess risk, yielding mutually exclusive burden estimates for each pathogen–drug combination.

### AMR forecasts

This study employed the ARIMA model to estimate AMR-related deaths, DALYs, and ASR projected for 2050 under the baseline scenario. The ARIMA model is a classic statistical tool for time series modelling and prediction, which combines autoregressive (AR), differencing (I) and moving average (MA) modules to capture temporal data characteristics. Model specification is presented as ARIMA (p,d,q), in which p is the autoregressive order, d is the number of differencing operations for time series stationarization, and q is the moving average order. Specifically, the AR term reflects linear associations between the current data point and its p lagged values, while the MA term accounts for linear combinations of the current observation and q prior residuals. The differencing procedure removes secular trends through *d*-order transformation to convert non-stationary sequences into stationary ones (Alabdulrazzaq et al., 2021). The core formula of the ARIMA model is as follows:


$$y_{t}=c+\phi_{1}y_{t-1}+\phi_{2}y_{t-2}+\cdots +\phi_{p}y_{t-p}+\theta_{1}\in_{t-1}+\theta_{2}\in_{t-2}+\cdots +\theta_{q}\in_{t-q}+\in_{t}.$$

Modulation of the three parameters allows the model to fit various time series and generate long-term forecasts. All modelling procedures were performed in R 4.3.3 with the forecast package (version 8.23.0).

## Results

### Global burden and trends of childhood sepsis from 1990 to 2021

Globally, the burden of pathogen-related sepsis in children aged <15 years declined substantially between 1990 and 2021, with consistent reductions in mortality, DALYs, ASMR, and ASDR. Sepsis deaths decreased from 6.43 million (95% UI, 4.96–7.90 million) in 1990 to 2.24 million (95% UI, 1.53–2.95 million) in 2021, while sepsis-related DALYs fell from 578 million (446–709 million) to 203 million (95% UI, 139–267 million). The annual percent change in ASMR (EAPC −3.35, 95% CI, −3.50 to −3.20) and ASDR (EAPC −3.33, 95% CI, −3.47 to −3.18) confirmed a sustained, moderate decline over three decades, aligning with global progress in childhood infectious disease control. AMR-related sepsis burden followed a parallel downward trajectory. Total AMR-attributable deaths declined from 516,700 (95% UI, 345,800–688,300) to 209,300 (95% UI, 136,700–282,900), and AMR-associated deaths from 2.44 million (95% UI, 1.72–3.16 million) to 918,700 (95% UI, 611,600–1,225,800). Corresponding reductions were observed for DALYs, ASMR, and ASDR (**Supplementary Tables 2**, **3**).

### Burden of childhood sepsis in super-regions in 2021

In 2021, childhood sepsis burden displayed pronounced and persistent interregional disparities, with Sub-Saharan Africa and South Asia bearing the vast majority of mortality, while high-income regions experienced minimal burden—a pattern consistent with global inequalities in healthcare access and socioeconomic conditions. Sub-Saharan Africa had the highest number of deaths at approximately 1,266,268 (95% UI, 807,566–1,725,459), followed by South Asia with 608,708 deaths (95% UI, 384,997–832,496). High-income regions had the fewest deaths, at only 10,588 (95% UI, 7,908–13,269) (**Table 1**). This disparity was reflected in the ASMR, with Sub-Saharan Africa having the highest rate at 251.40 per 100,000 (95% UI, 160.17–342.73), compared to 6.94 per 100,000 in High-income regions (95% UI, 5.16–8.72). A similar pattern was observed for DALYs, with the greatest burden in Sub-Saharan Africa and South Asia, and the lowest in High-income regions (**Table 1**). AMR-related sepsis further amplified these regional gaps, reflecting limited access to effective antibiotics, diagnostics, and infection control measures in low-resource settings. For AMR-related childhood sepsis deaths, Sub-Saharan Africa also had the highest number at 588,524 (95% UI, 363,979–813,929), followed by South Asia with 356,440 deaths (95% UI, 222,844–490,852). High-income regions reported only 3,554 deaths (95% UI, 2,724–4,385) (**Supplementary Table 1**). Regional differences in DALYs due to AMR followed a similar trend (**Supplementary Table 1**).

**Table 1:** Global and super-regional sepsis disease burden caused by pathogenic bacteria and their changing trends from 1990 to 2021.

Location	Counts (95% UI)		ASR per 100,000 (95% UI)		EAPCs (95% CI)
	1990	2021	1990	2021	
Deaths					
Global	6429991 (4963675, 7896750)	2240140 (1533739, 2947092)	357.78 (276.07, 439.52)	122.79 (84.11, 161.50)	-3.35 (-3.50, -3.20)
Central Europe, Eastern Europe, and Central Asia	120442 (103212, 137686)	26369 (20012, 32726)	125.75 (107.61, 143.91)	37.59 (28.46, 46.72)	-4.26 (-4.53, -4.00)
High-income	35946 (28669, 43223)	10588 (7908, 13269)	20.37 (16.23, 24.51)	6.94 (5.16, 8.72)	-3.04 (-3.16, -2.92)
Latin America and Caribbean	319876 (273942, 365851)	66322 (46775, 85874)	224.40 (192.22, 256.61)	50.19 (35.35, 65.04)	-4.38 (-4.53, -4.24)
North Africa and Middle East	407115 (291200, 523149)	93536 (61232, 125848)	275.23 (196.90, 353.63)	55.27 (36.14, 74.42)	-4.84 (-4.97, -4.71)
South Asia	2273213 (1641826, 2904732)	608708 (384997, 832496)	498.21 (359.41, 637.04)	136.54 (86.42, 186.68)	-3.90 (-4.04, -3.76)
Southeast Asia, East Asia, and Oceania	1237988 (933083, 1543236)	167575 (116812, 218423)	247.48 (186.54, 308.49)	48.27 (33.66, 62.92)	-5.60 (-5.74, -5.45)
Sub-Saharan Africa	2033514 (1470464, 2597078)	1266268 (807566, 1725459)	743.68 (535.88, 951.66)	251.40 (160.17, 342.73)	-3.31 (-3.50, -3.12)
DALYs					
Global	577725080 (446004410, 709494298)	202817533 (138589284, 267096131)	32142.75 (24803.05, 39485.19)	11111.79 (7599.60, 14626.69)	-3.33 (-3.47, -3.18)
Central Europe, Eastern Europe, and Central Asia	10903332 (9335025, 12473351)	2392938 (1812730, 2973189)	11383.24 (9733.48, 13034.76)	3409.79 (2578.35, 4241.29)	-4.25 (-4.51, -3.99)
High-income	3431275 (2680884, 4181667)	1053474 (772157, 1334792)	1941.12 (1516.94, 2365.29)	684.08 (500.83, 867.34)	-2.91 (-3.02, -2.80)
Latin America and Caribbean	28876654 (24710670, 33046687)	6011742 (4237667, 7786607)	20257.79 (17339.50, 23178.90)	4548.20 (3201.86, 5895.13)	-4.38 (-4.52, -4.24)
North Africa and Middle East	36684415 (26208493, 47171883)	8513428 (5530296, 11497894)	24797.02 (17717.20, 31884.63)	5027.72 (3264.37, 6791.83)	-4.81 (-4.94, -4.68)
South Asia	203940369 (147386763, 260516367)	55533459 (34955229, 76123702)	44684.33 (32256.47, 57117.18)	12434.73 (7839.93, 17032.08)	-3.85 (-3.99, -3.72)
Southeast Asia, East Asia, and Oceania	111449964 (83923970, 139004136)	15330101 (10651605, 20014903)	22280.62 (16778.81, 27788.08)	4407.04 (3065.99, 5749.86)	-5.56 (-5.70, -5.41)
Sub-Saharan Africa	182269562 (131754969, 232831787)	113912431 (72559860, 155316738)	66614.24 (47974.10, 85271.79)	22616.40 (14389.18, 30853.82)	-3.30 (-3.49, -3.11)

ASR, age-standardized rates; DALYs, disability-adjusted life-years; UI, uncertainty interval; CI, confidence interval.

### Top pathogens contributing to childhood sepsis burden in 2021

**Figure 1:**
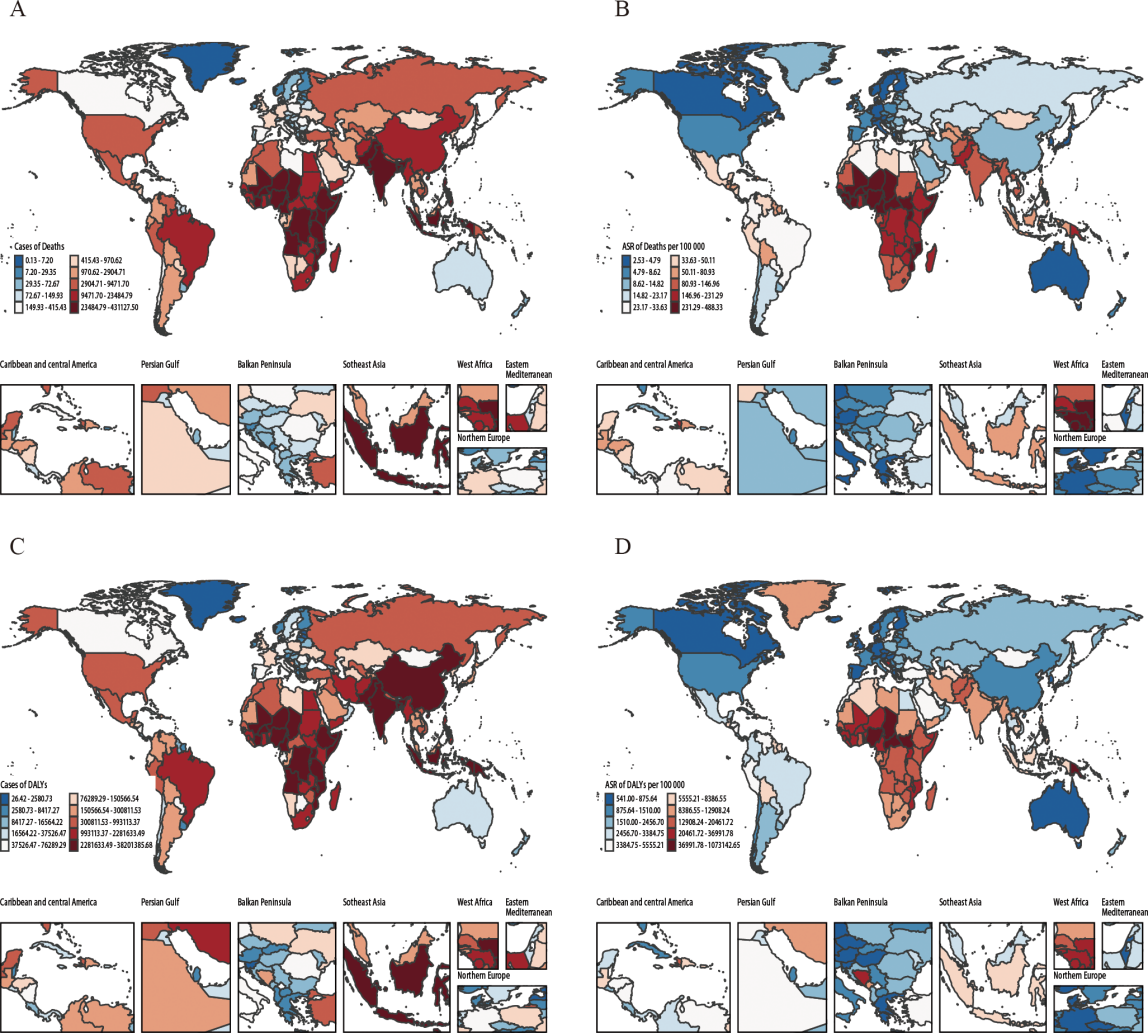
Global burden of pathogen-associated sepsis in children across 204 countries and territories in 2021. **(A)** Death cases, **(B)** Age-standardized mortality rate (ASMR), **(C)** Disability-adjusted life year (DALY) cases, **(D)** Age-standardized DALY rate (ASDR). DALY, disability-adjusted life-year; ASMR, age-standardized mortality rate; ASDR, age-standardized disability-adjusted life year rate.

Streptococcus pneumoniae, Klebsiella pneumoniae, Escherichia coli, Pseudomonas aeruginosa, and Staphylococcus aureus dominated the global pathogen spectrum of childhood sepsis in 2021, collectively responsible for over half of sepsis-related deaths. S. pneumoniae and K. pneumoniae each caused >100,000 deaths, and E. coli nearly 100,000 deaths (**Supplementary Table 4**). Notable regional heterogeneity existed in leading pathogens: S. pneumoniae predominated in four super-regions, K. pneumoniae in Latin America/Caribbean and South Asia, and S. aureus in high-income regions (**Supplementary Figure 1**), consistent with region-specific pathogen ecology and healthcare practices. Country-level burden was concentrated in low-income nations of Sub-Saharan Africa and South Asia, consistent with high population vulnerability and limited healthcare infrastructure. Countries with the highest number of pathogen-related child deaths were mainly concentrated in Sub-Saharan Africa and South Asia. Nigeria, India, and Palestine each reported more than 150,000 child deaths. In terms of ASMR, the burden was most severe in Sub-Saharan African countries. Chad, South Sudan, and the Central African Republic had rates exceeding 450 per 100,000 population. Regarding DALYs, the countries with the highest pathogen-related burden were also concentrated in Sub-Saharan Africa and South Asia, with Nigeria, India, and Palestine again ranking top three. However, the countries with the highest ASDR were primarily located in Oceania (**Figure 1**).

### Ranking of childhood sepsis burden due to AMR in 2021

**Figure 2:**
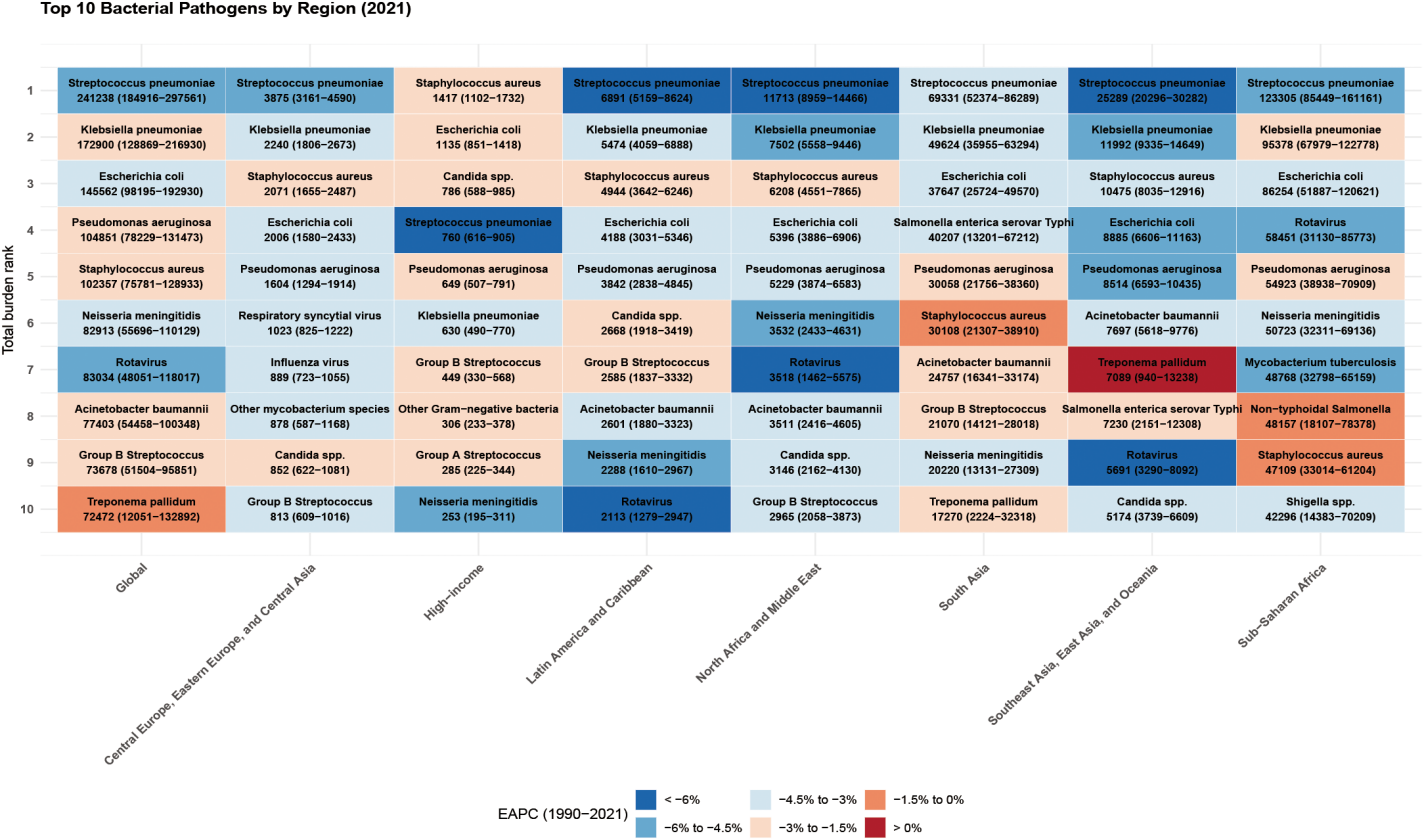
Top ten deadliest pathogen-drug combinations linked to AMR burden in children at the global and super-region levels, 2021. Cell colors indicate the estimated annual percentage change (EAPC) during 1990–2021, and the total number of child deaths in 2021 is annotated at the bottom of each cell. AMR, antimicrobial resistance; EAPC, estimated annual percentage change.

For AMR-attributable deaths, the top five pathogens were S. pneumoniae, K. pneumoniae, E. coli, Acinetobacter baumannii, and S. aureus, each linked to ≥100,000 annual deaths (**Figure 2**). While S. pneumoniae led mortality in most regions, S. aureus was predominant in high-income settings. Treponema pallidum (syphilis) burden increased in Southeast/East Asia and Oceania (EAPC >0%), contrasting with widespread declines in other AMR pathogens; S. pneumoniae exhibited the most pronounced reduction, likely driven by vaccine scale-up. When stratified by AMR attribution, K. pneumoniae emerged as the leading cause of directly attributable deaths (39,502; 95% UI, 28,662–50,343), while S. pneumoniae dominated associated deaths (192,503; 95% UI, 140,341–446,658) (**Supplementary Figure 2**).

### Burden of AMR-related sepsis in children across age groups

**Figure 3:**
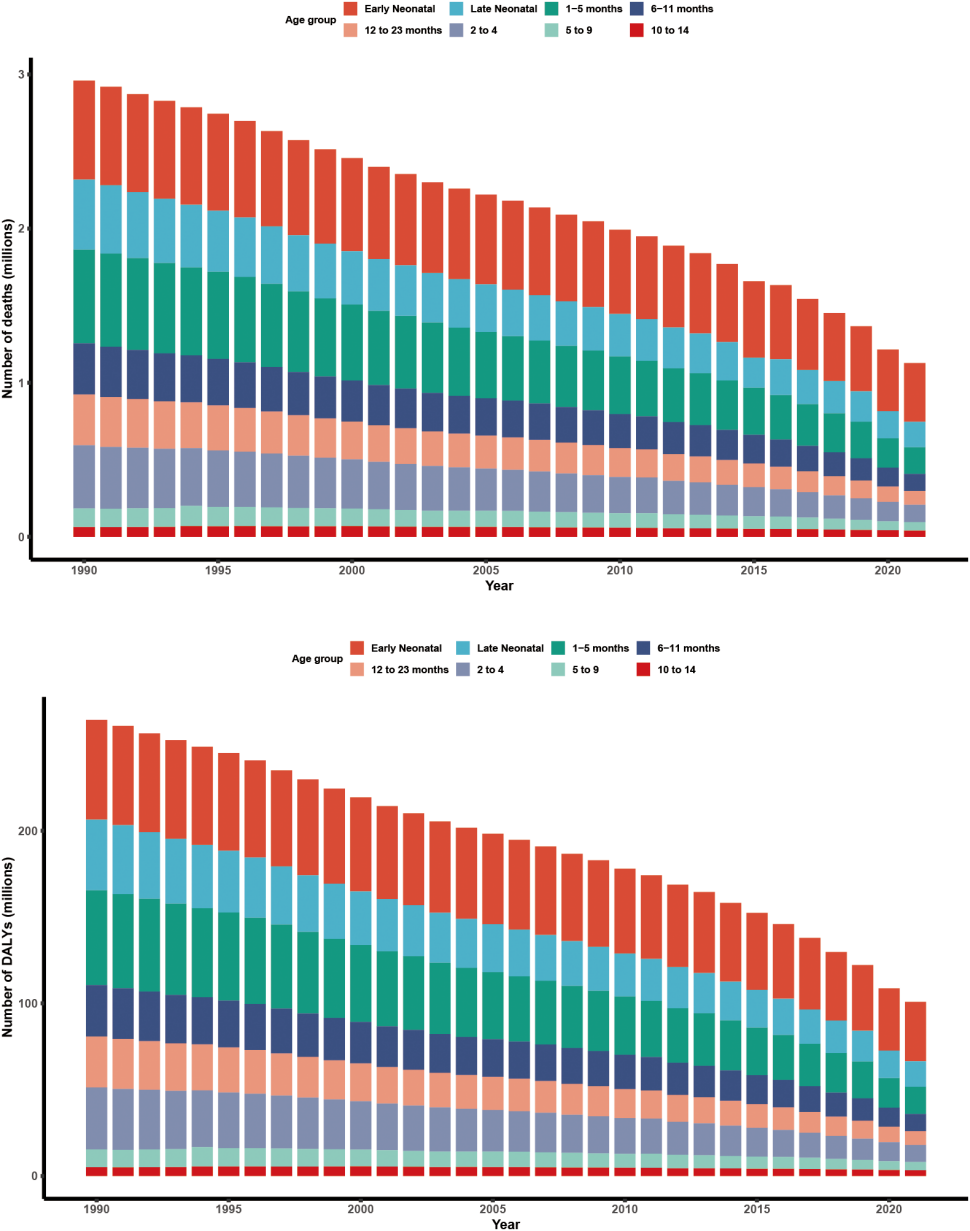
Long-term global trends in the burden of pathogen-associated pediatric sepsis across all age groups from 1990 to 2021.

Age distribution of AMR-related sepsis mortality was strongly skewed toward early life, with neonates and young infants at the highest risk throughout the study period. In 1990, early neonates had the highest deaths (641,700; 95% UI, 456,600–826,800), followed by infants aged 1–5 months (609,900; 95% UI, 451,200–769,000). By 2021, mortality declined across all ages but remained concentrated in early life: early neonates (381,900; 95% UI, 245,100–518,700) and 1–5-month-olds (174,300; 95% UI, 130,000–218,800) still accounted for >70% of AMR-related deaths, with the lowest burden in children aged 10–14 years (41,200; 95% UI, 27,300–55,200) (**Figure 3**, **Supplementary Table 1**). Pathogen distribution also varied by age: K. pneumoniae dominated neonatal sepsis, S. pneumoniae predominated in infants aged 1–5 months to 2–4 years, and Salmonella typhi was the leading pathogen in children ≥5 years (**Supplementary Figures 4**, **5**). DALY burden mirrored mortality patterns (**Supplementary Figures 6**, **7**).

### Ranking of pathogen-associated sepsis syndromes in childhood in 2021

**Figure 4:**
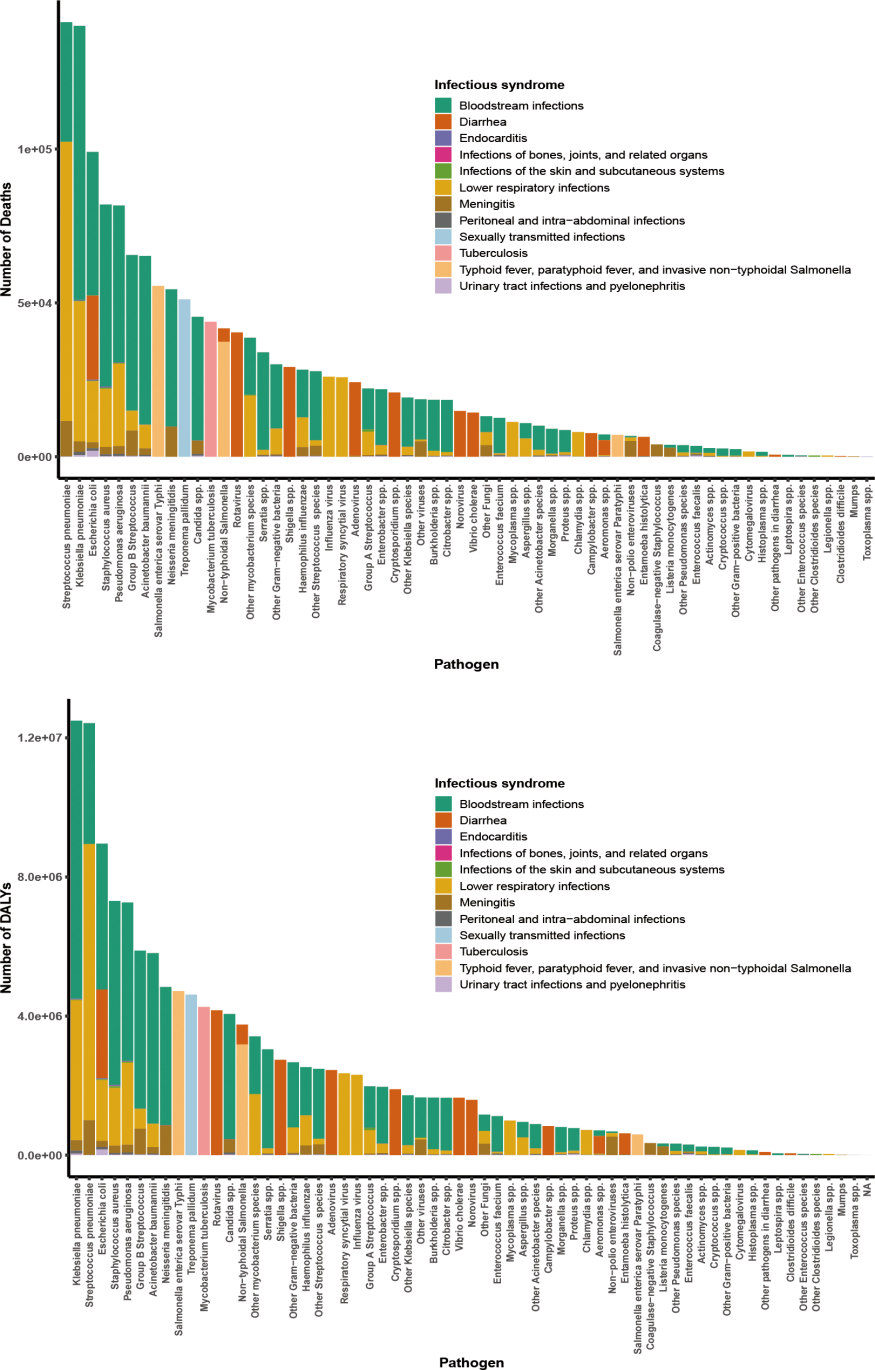
Global burden of childhood infectious diseases attributable to distinct pathogens and infectious syndromes in 2021. Each column represents the overall death or DALY count of a specific pathogen, with segmented sections corresponding to different infectious syndrome subtypes. DALY, disability-adjusted life-year.

In 2021, bloodstream infections, lower respiratory infections, and diarrheal diseases collectively accounted for >80% of pathogen-related childhood sepsis deaths, representing the dominant clinical syndromes globally. Bloodstream infections caused the most deaths (852,484), driven by K. pneumoniae (89,112 deaths) and S. aureus (59,181 deaths). Lower respiratory infections resulted in 593,739 deaths, primarily due to S. pneumoniae (90,727 deaths) and K. pneumoniae (45,552 deaths). Diarrheal diseases led to 387,370 deaths, with rotavirus as the leading pathogen (40,369 deaths) (**Figure 4**, **Supplementary Figure 8**). Bloodstream infections had the highest ASMR (47.62 per 100,000), followed by lower respiratory infections (32.41) and diarrhea (21.08), highlighting the critical role of invasive bacterial disease in childhood sepsis mortality (**Supplementary Table 5**).

### AMR burden forecasts

**Figure 5:**
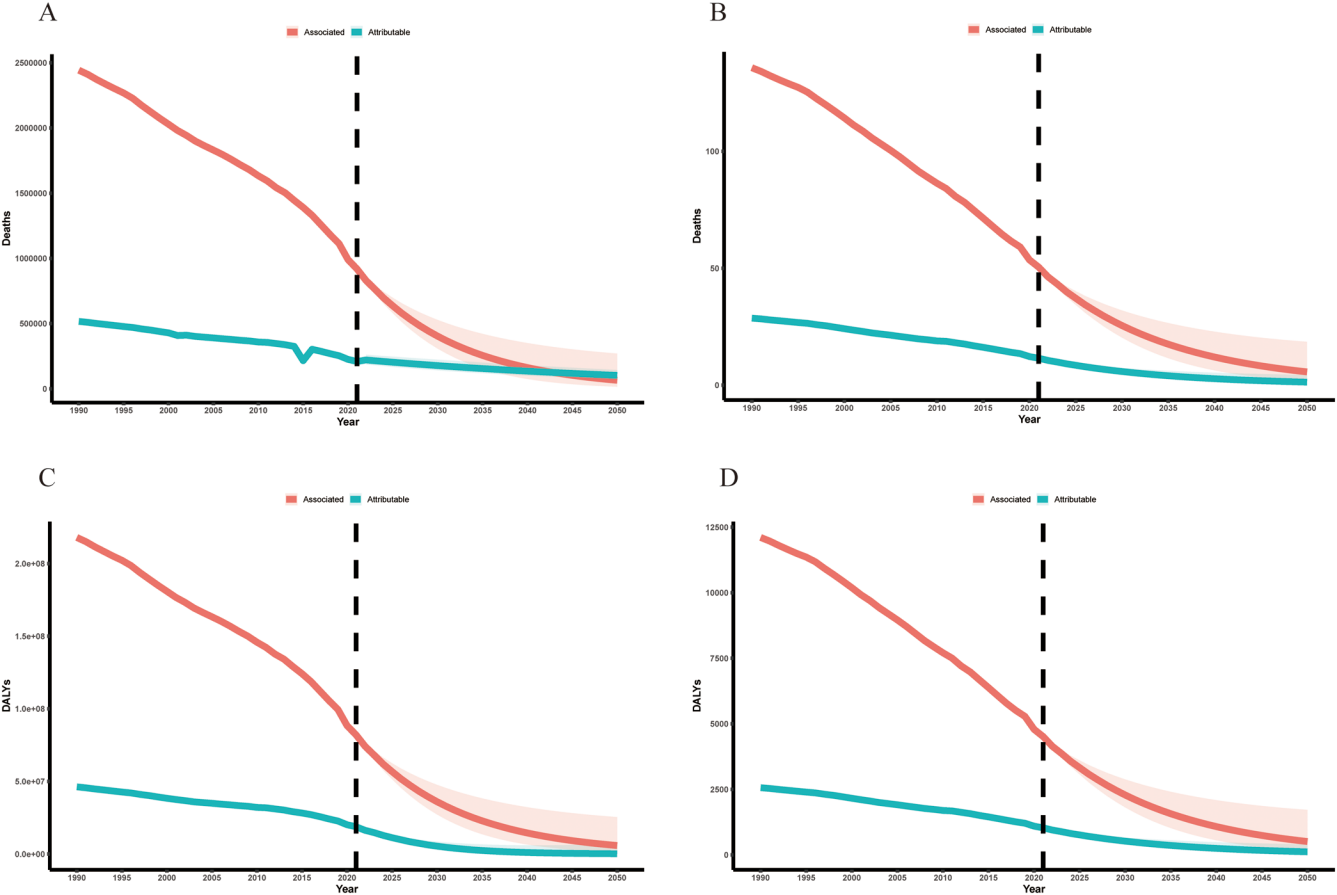
Projected global burden of childhood diseases associated with and attributable to AMR under the reference scenario, 2022–2050. **(A)** Number of AMR-associated deaths; **(B)** AMR-associated death rate; **(C)** Number of AMR-attributable DALYs; **(D)** AMR-attributable DALY rate. Shaded areas represent 95% uncertainty intervals. The vertical dividing line at 2021 distinguishes historical observational estimates from future predictive forecasts. AMR, antimicrobial resistance; DALY, disability-adjusted life-year.

Under current trends and plausible intervention scenarios, the global burden of AMR-related childhood sepsis is projected to continue declining through 2050, though substantial uncertainty surrounds these estimates (**Figure 5**). By 2050, AMR-attributable child deaths are projected to decrease to approximately 103,080 (95% UI, 73,530–144,505), and AMR-associated deaths to 65,362 (95% UI, 15,807–270,272) (**Supplementary Table 6**). The projected ASMR for attributable and associated deaths would decline to 1.31 (95% UI, 0.39–4.40) and 5.67 (95% UI, 1.74–18.55) per 100,000, respectively, reflecting anticipated gains in vaccination, antimicrobial stewardship, and pediatric care. Corresponding reductions are expected for DALYs, though wide uncertainty intervals underscore the conditional nature of these projections (**Supplementary Tables 6**, **7**).

## Discussion

This study systematically analyzed the global burden of pathogen- and AMR-related childhood sepsis from 1990 to 2021 and projected future trends. Results showed significant reductions in child deaths, DALYs, ASMR, and ASDR associated with sepsis, consistent with previous research and indicating that global prevention and control efforts have been effective (Rudd et al., 2020). This progress is largely attributed to action plans implemented by governments and international organizations to address pathogens and AMR (Pallett et al., 2023; WHO, 2023). Nevertheless, despite overall reductions, sepsis remains a major threat to child health globally, especially in low- and middle-income countries (LMICs).

Although high-income regions witness high antibiotic consumption, the burden of childhood sepsis is heavily concentrated in sub-Saharan Africa and South Asia, while such burden remains extremely low in high-income nations. This striking disparity reflects long-standing imbalances in global healthcare accessibility, diagnostic capacity and socioeconomic development. In low- and middle-income countries, underdeveloped laboratory infrastructure hampers microbial detection and evidence-based clinical management, triggering inappropriate antibiotic use (WHO, 2018). Loose regulatory oversight, inadequate supply of second- and third-line antibiotics (Holmes et al., 2016), proliferation of counterfeit pharmaceuticals (Kelesidis and Falagas, 2015) and poor sanitary conditions further escalate antimicrobial resistance risks (Ramay et al., 2020). Merely 15% of WHO African Region member states conduct routine antimicrobial resistance surveillance (Leopold et al., 2014). Coupled with high infection prevalence, over-the-counter antibiotic access and agricultural antibiotic misuse, these factors facilitate the widespread dissemination of ESBL-producing and other multidrug-resistant organisms (Eagar et al., 2012; Kissoon and Uyeki, 2016). South Asia bears an overwhelming burden of neonatal sepsis, predominantly attributable to poverty, low hospital delivery rates, inequitable resource allocation and substandard hygiene in perinatal and neonatal care (Das et al., 2015).

A major innovation of this study lies in the age- and region-stratified mapping of sepsis pathogen spectra, which reveals systematic disparities in predominant pathogens across diverse populations. Multiple licensed commercial Salmonella Typhi vaccines have been proven effective in reducing childhood typhoid infection, severe sepsis and mortality, serving as a core intervention against Salmonella infection among older children (Gloeck et al., 2025). Nevertheless, Salmonella Typhi remains the leading lethal pathogen in children aged 5 years and above, pointing to prominent deficiencies in global vaccine coverage, vaccination timing and promotion strategies (Hancuh et al., 2023; Shakya and Pollard, 2024). Klebsiella pneumoniae constitutes the predominant pathogen of neonatal sepsis in South Asia and Latin America, with its prevalence closely linked to antibiotic overuse and resistant strain transmission (Falagas et al., 2025). Following the widespread rollout of pneumococcal and *Haemophilus influenzae* type b vaccines, *Staphylococcus aureus* has emerged as the primary invasive pathogen in high-income countries (Kourtis et al., 2019), with its incidence exceeding the historical levels prior to vaccine implementation (Spaulding et al., 2018; Proctor, 2021). Despite the universal empirical antibiotic administration guidelines issued in the WHO Model List of Essential Medicines, the findings strongly advocate for age- and region-specific clinical protocols to curb irrational antibiotic use and restrict the spread of resistant bacteria in low- and middle-income countries (Kourtis et al., 2019). A three-tiered targeted intervention framework is hereby proposed. First, high-income regions should prioritize staphylococcal infection prevention and post-vaccination pathogen surveillance. Second, sub-Saharan Africa needs to scale up point-of-care testing and restrict over-the-counter antibiotic sales in community pharmacies. Third, South Asia shall strengthen nosocomial infection control in delivery rooms and implement carbapenem stewardship to contain *Klebsiella pneumoniae* resistance. Such targeted strategies can effectively reduce inappropriate antibiotic utilization and slow antimicrobial resistance progression.

The study further identifies five WHO priority pathogens as the major drivers of drug-resistant childhood sepsis, with divergent epidemiological trends (WHO, 2023; WHO, 2024). Pneumococcal disease burden has declined substantially owing to PCV deployment, whereas syphilis-associated sepsis has risen in Southeast Asia, indicative of insufficient preventive measures and improper antibiotic administration (Newman et al., 2013). Vaccination stays the cornerstone strategy for pneumococcal infection control (WHO, 2019). Vaccine development remains urgently needed for high-burden pathogens lacking licensed vaccines, such as *Staphylococcus aureus* and *Escherichia coli (*Jansen et al., 2018*)*. Among children aged 2 to 4 years in low- and middle-income countries, PCV reduces antibiotic-treated lower respiratory tract infections by 20% (Lewnard et al., 2020a) and alleviates multidrug antimicrobial resistance (Andrejko et al., 2021).

In terms of disease categories, bloodstream infections, lower respiratory tract infections and diarrheal diseases collectively account for over 80% of sepsis-related deaths, with bloodstream infections constituting an underestimated yet critical intervention target (WHO, 2020b). *Klebsiella pneumoniae* is the predominant causative agent of bloodstream infections and contributes to substantial mortality, and carbapenem-resistant strains spread extensively alongside increased neonatal antibiotic exposure (Hu et al., 2023). Pneumococcal vaccines prevent approximately 250,000 deaths annually, yet pneumococci still rank first among pathogens causing fatal lower respiratory tract infections in children (Wahl et al., 2018). Rotavirus remains the leading pathogen responsible for severe pediatric diarrhea, and a large proportion of diarrheal cases in low- and middle-income countries receive unnecessary antibiotic treatment (Lewnard et al., 2020b; Brennhofer et al., 2022). Rotavirus vaccines cut antibiotic-treated diarrheal episodes by 11% in children under 2 years old (Lewnard et al., 2020a). Immunization programs and public health initiatives have successfully lowered mortality rates, and further progress relies on expanded vaccine coverage and stringent antibiotic stewardship, particularly in resource-limited settings.

This study conducts long-term projections of drug-resistant childhood sepsis burden up to 2050. The results suggest that universal vaccination, standardized antibiotic management and strengthened infection control will steadily reduce the disease burden (Mendelson et al., 2024). Notably, the reduction in overall resistance-attributable burden outpaces that directly caused by resistant pathogens, demonstrating that public health interventions including sanitation improvement and standardized clinical procedures can mitigate indirect hazards of resistant infections. Even so, an estimated 100,000 children will still die annually from bacterial antimicrobial resistance by the mid-century. Continuous investment is therefore required for novel antimicrobial drug research, exploration of resistance gene transmission mechanisms and containment of resistant pathogen spread (Lewnard et al., 2024).

This study has several limitations. First, analyses based on the GBD 2021 database are subject to its inherent methodological limitations and systematic biases. Owing to imperfect surveillance systems in LMICs, relevant data rely heavily on model extrapolation, which tends to underestimate the regional burden of sepsis and AMR infections. Additionally, the globally unified diagnostic criteria fail to accommodate the heterogeneous clinical practices and microbial testing capacities across regions (Collaborators GAR, 2024). Second, data on infections and antimicrobial resistance remain extremely scarce and fragmented in low-income countries, with poor representativeness, a situation attributable to inadequate laboratory infrastructure and healthcare resources (Collaborators GAR, 2022; Collaborators GAR, 2024). Furthermore, the sampling frameworks of conventional population surveys cannot accurately reflect the real-world patterns of antimicrobial consumption (Browne et al., 2021). Third, the predictions in this study are generated based on historical trends, and thus cannot account for the impacts of emerging resistance determinants and public health emergencies on the transmission of drug-resistant pathogens. While COVID-19 containment measures temporarily curbed the spread of resistant pathogens, they disrupted routine microbial surveillance, resulting in data gaps and sampling biases during 2020–2021 (Subramanya et al., 2021; Li et al., 2024). These issues may compromise the accuracy of model-based projections for AMR evolution in the post-pandemic era.

## Conclusion

Despite several limitations, this study provides the most systematic and comprehensive global assessment of pediatric sepsis burden attributable to bacterial pathogens and AMR. Using GBD-validated models and multi-source epidemiological data, this study improved the accuracy of global estimates of AMR-associated pediatric sepsis. For the first time, we characterized the spatiotemporal patterns of multi-pathogen pediatric sepsis across 204 countries and regions, filling critical gaps in large-scale, long-term global evidence. AMR substantially endangers child health, with the highest pediatric risks concentrated in sub-Saharan Africa and South Asia. Key pathogens including *Streptococcus pneumoniae*, *Klebsiella pneumoniae*, *Escherichia coli*, and *Staphylococcus aureus* predominantly drive pediatric mortality via bloodstream, respiratory, and gastrointestinal infections. These findings support optimized pediatric infection management guidelines. Mitigating global AMR burden requires strengthened laboratory surveillance, improved healthcare systems, expanded preventive strategies, rational antimicrobial use, and advanced epidemiological trend research.

## Data Availability

The datasets presented in this study can be found in online repositories. The names of the repository/repositories and accession number(s) can be found in the article/**Supplementary Material**.
