# Supplementary material for: Childhood sepsis burden: pathogens/antimicrobial-resistant bacteria, 1990–2021 and 2050 forecasts
Source: Front Cell Infect Microbiol. 2026 Jun 19;16:1855912. doi: 10.3389/fcimb.2026.1855912 (PMC13328457; doi:10.3389/fcimb.2026.1855912)
Supplement: Supplementary file 1 [file Table1.docx]

FigS1. Global sepsis-related deaths (A) and DALYs (B) in children by pathogen and GBD super-region, 2021.

Data are presented for the 15 pathogens with the largest number of global deaths; the Others group comprises the additional 18 bacteria estimated in this study. GBD, Global Burden of Disease; DALYs, disability-adjusted life-years.


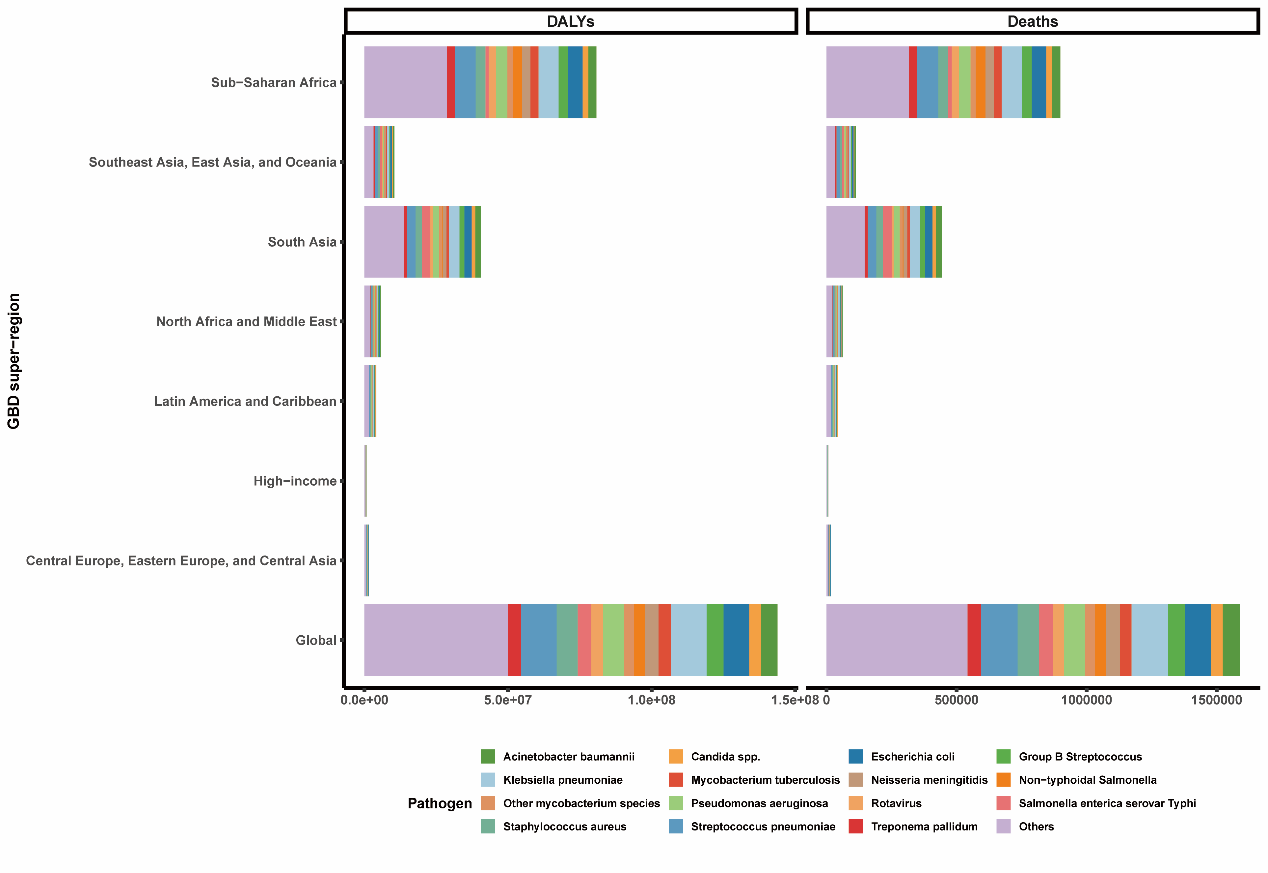


Fig S2. The 10 deadliest pathogen–drug combinations by AMR-attributable and AMR-associated disease burden, globally and by super-region, among children, 2021.

Cells are colored by the EAPC (1990–2021). The total number of deaths or DALYs in 2021 is presented at the bottom of each cell. AMR, antimicrobial resistance; EAPC, Estimated Annual Percentage Change; DALYs, disability-adjusted life-years.


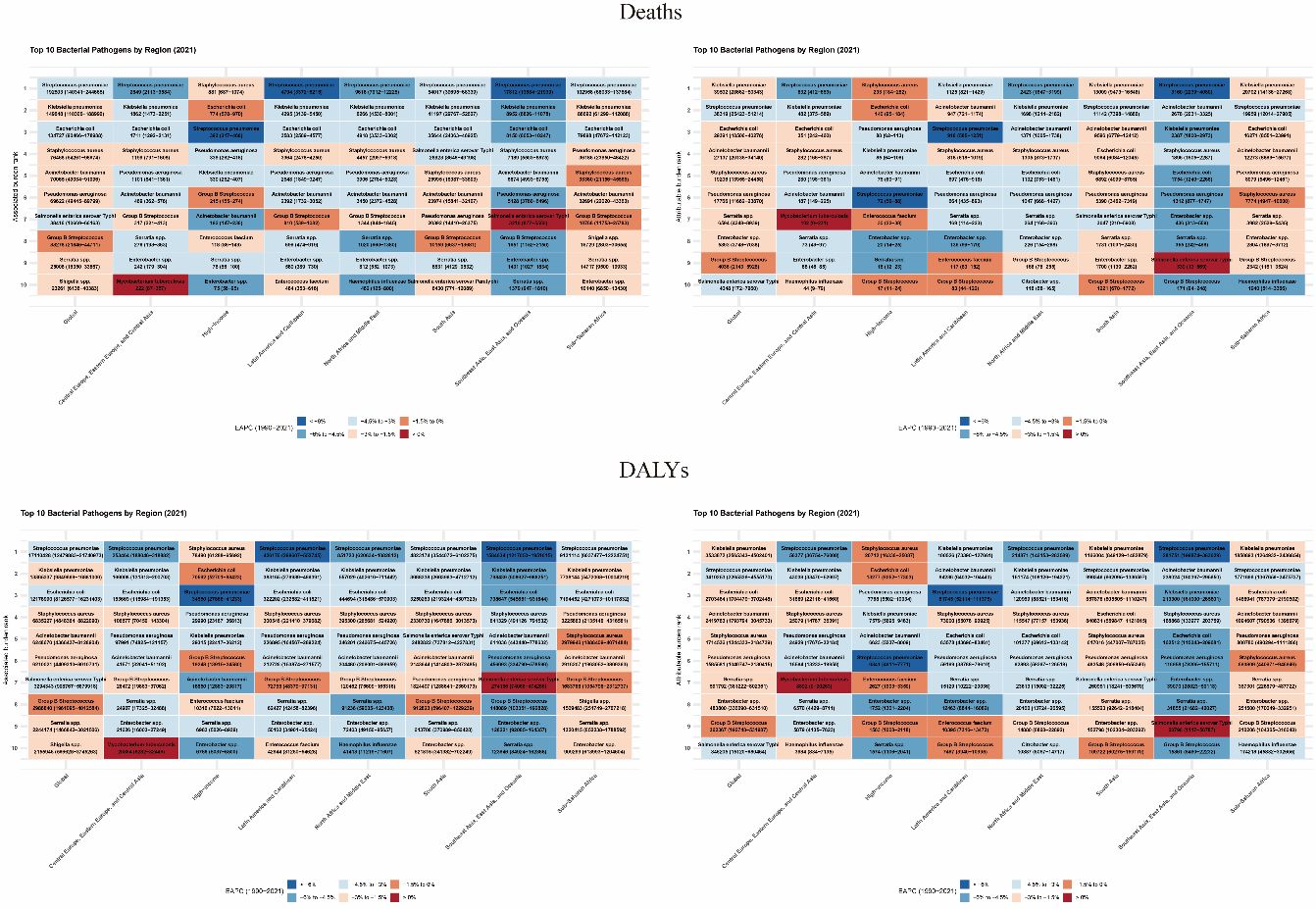


Fig S3 The top 10 high-DALY-burden pathogen–drug combinations, by burden associated with AMR, globally and super-region, among children, 2021.

Cells are colored by the EAPC (1990–2021). The total number of DALYs in 2021 is presented at the bottom of each cell. AMR, antimicrobial resistance; EAPC, Estimated Annual Percentage Change; DALYs, disability-adjusted life-years.


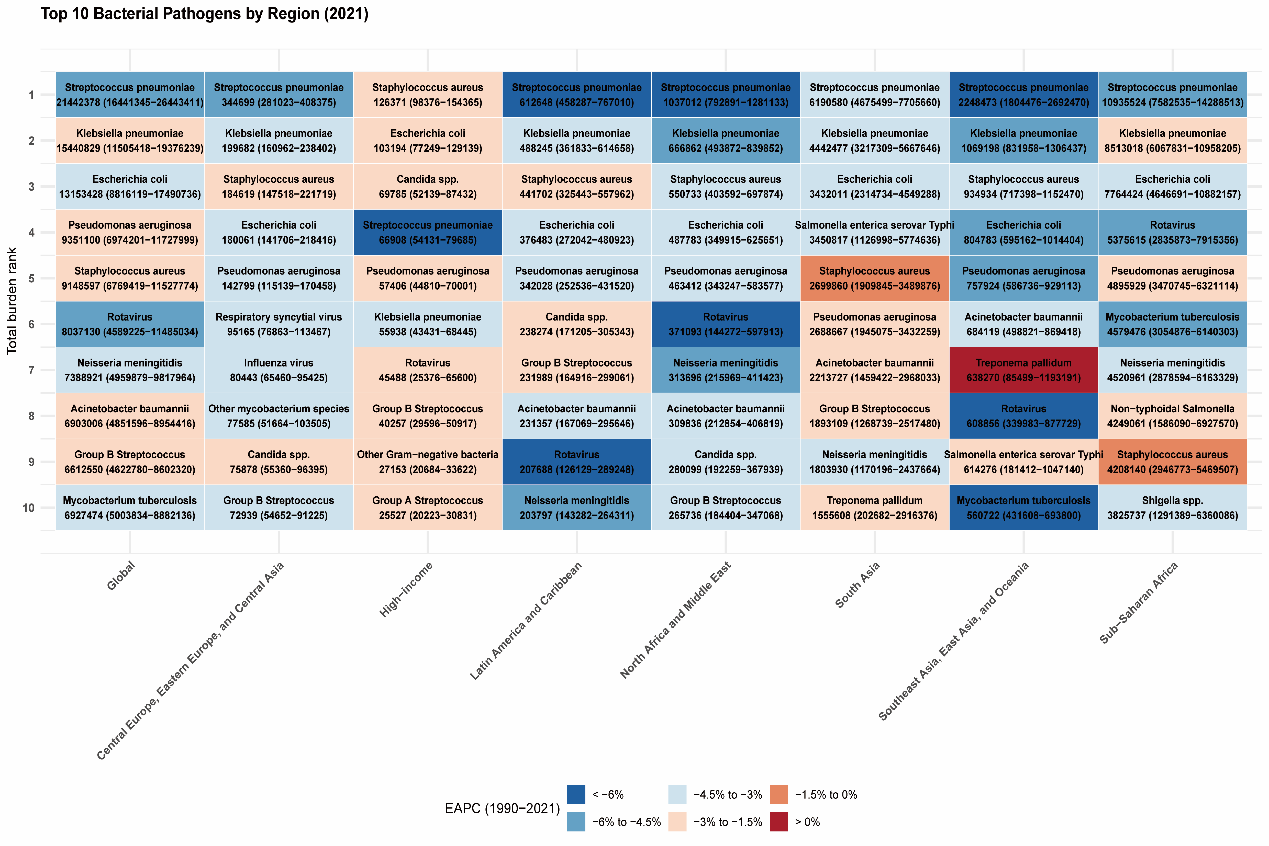


Fig S4 Global number of deaths by pathogen, age and sex group, 2021.


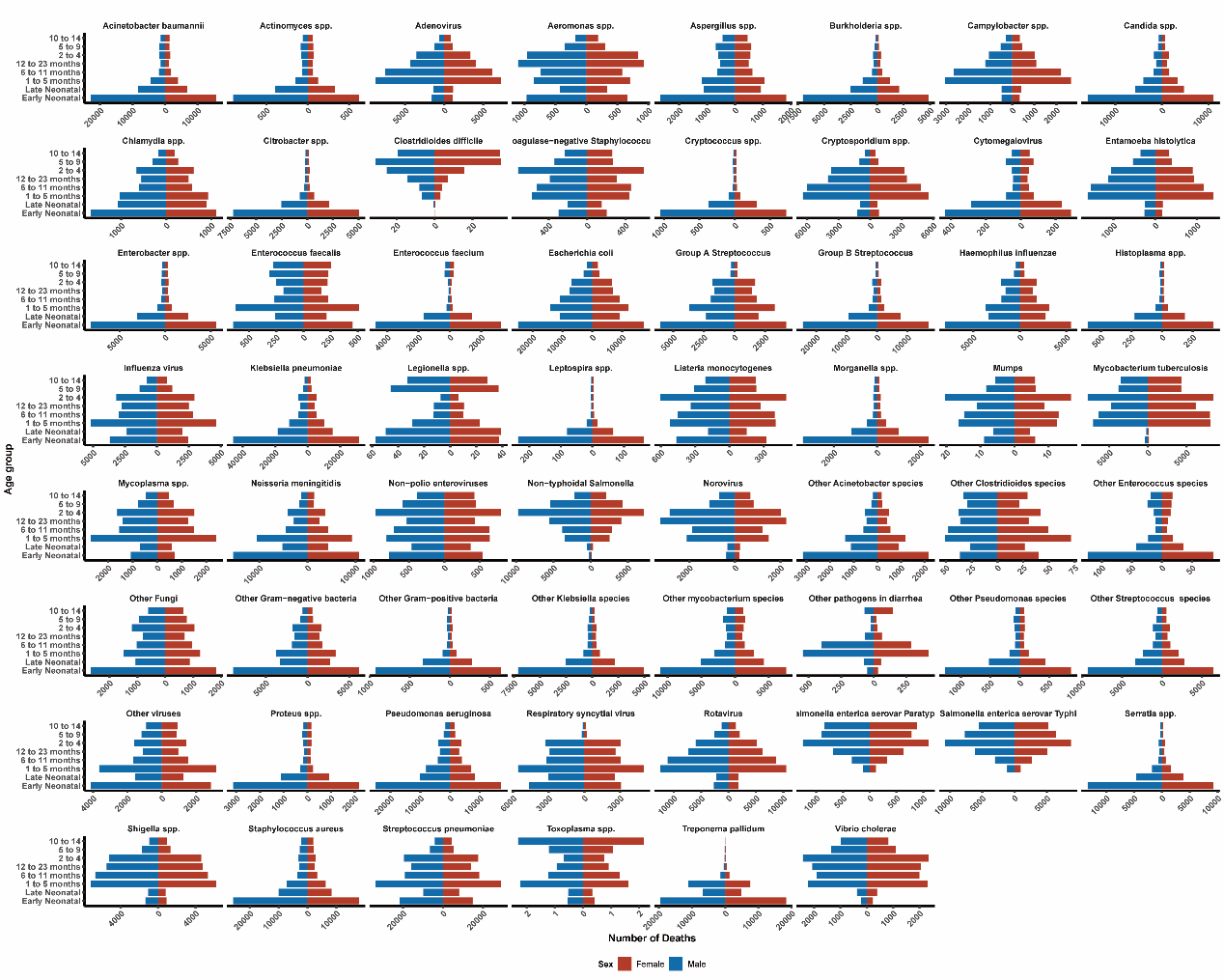


Fig S5 Global Death Rate by pathogen, age and sex group, 2021


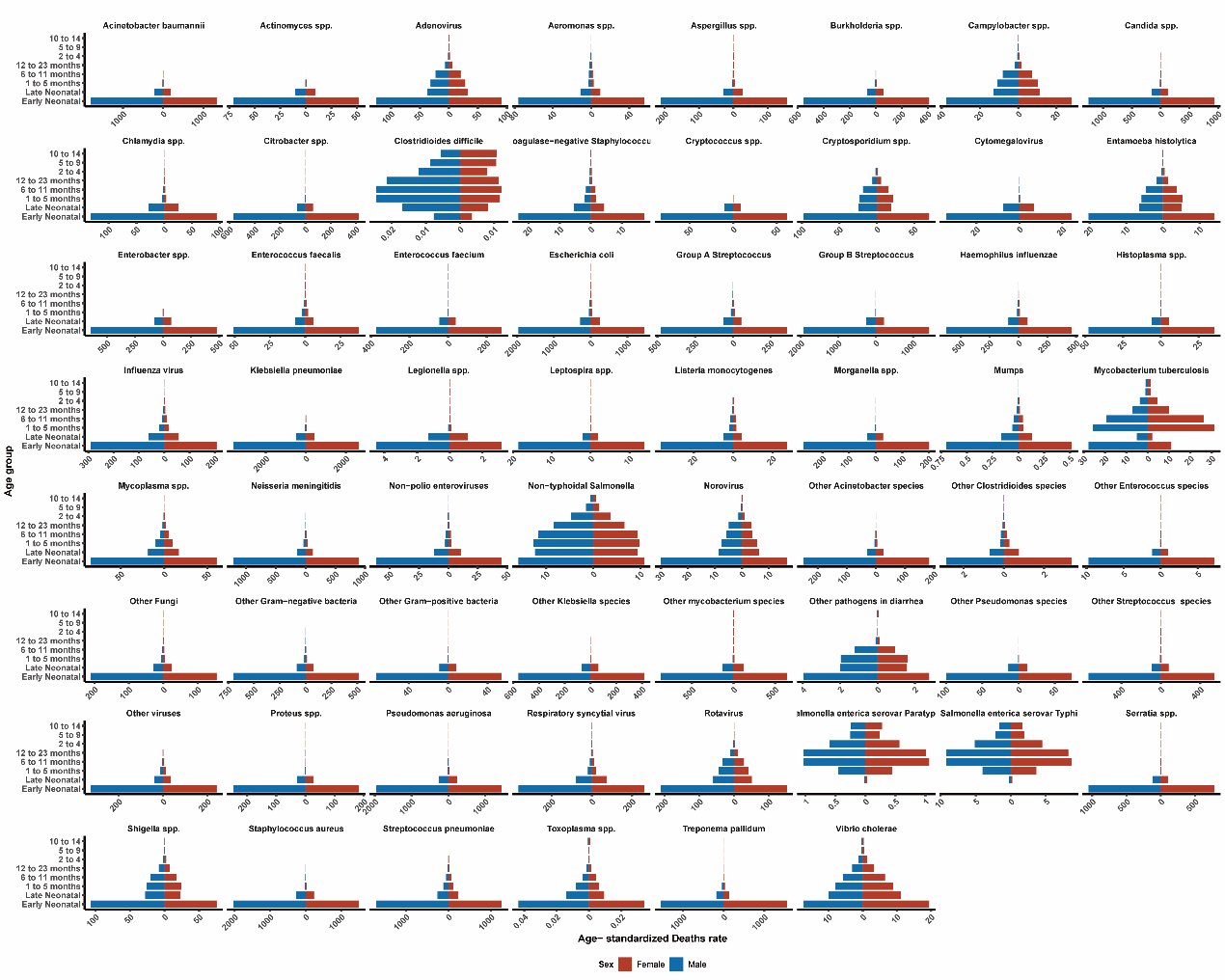


Fig S6 Global number of DALYs by pathogen, age and sex group, 2021. DALYs, disability-adjusted life-years.


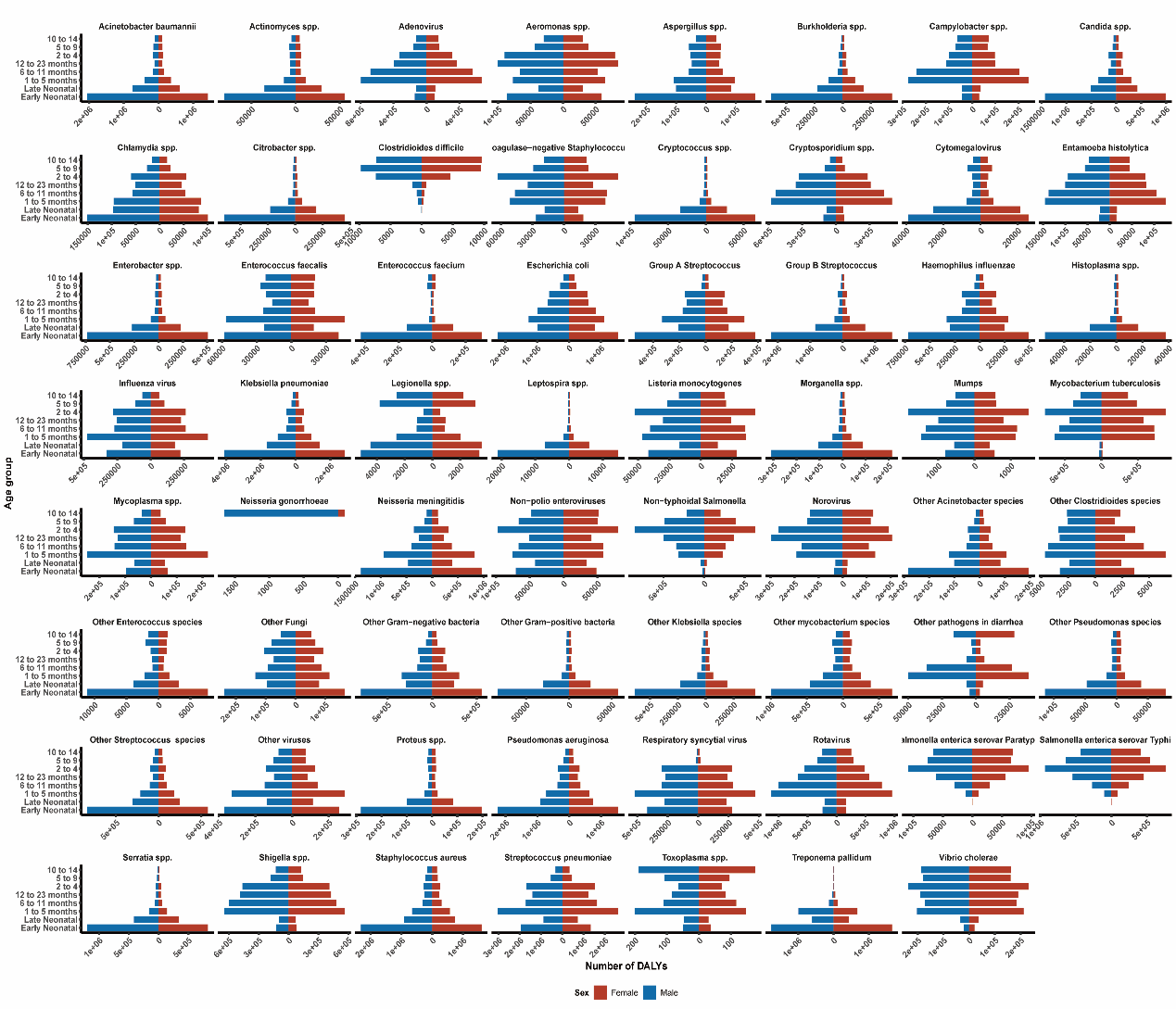


Figure S7 Global DALYs Rate by pathogen, age and sex group, 2021. DALYs, disability-adjusted life-years.


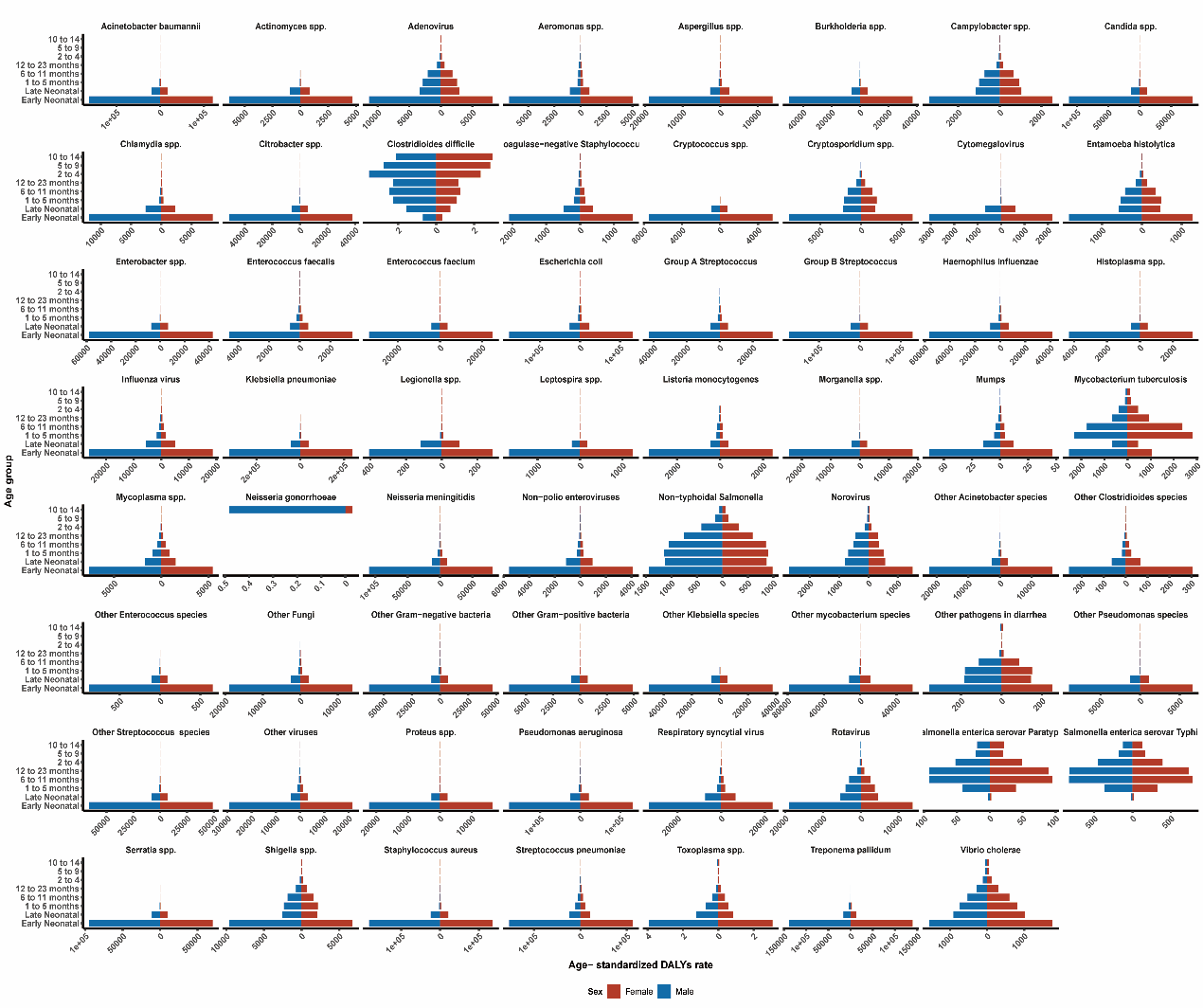


Figure S8 Global percentage of deaths and DALYs among children due to each infectious syndrome, 2021. DALYs, disability-adjusted life-years.


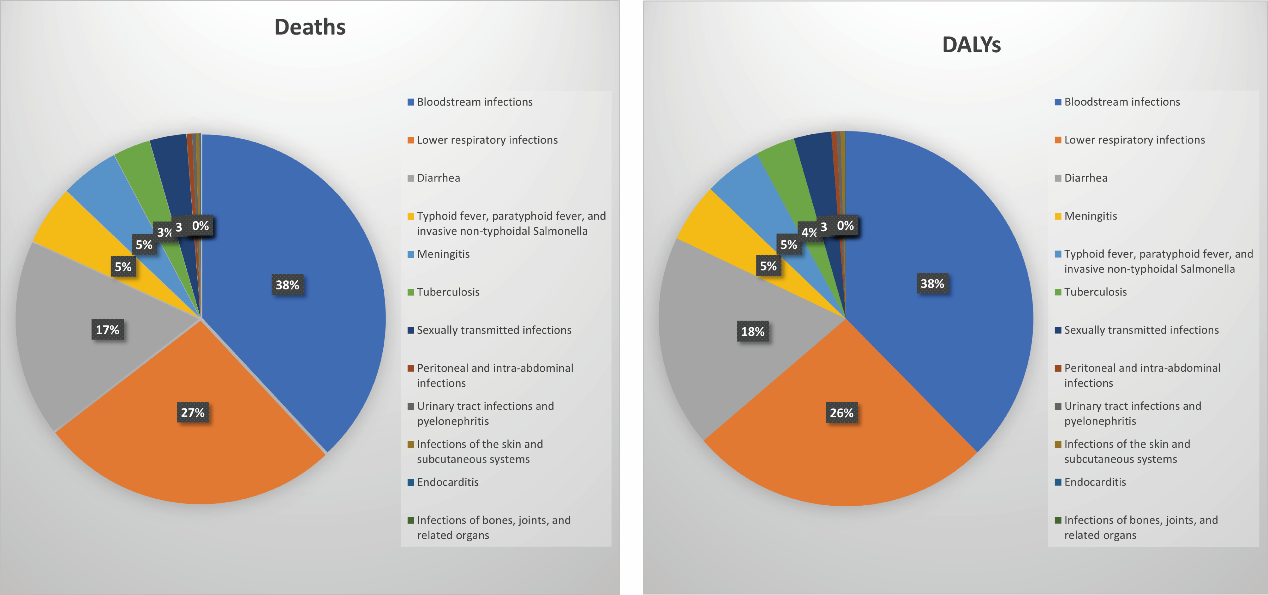


Table S1 Global, super-regional, and age-group-specific sepsis disease burden related to antimicrobial-resistant bacteria from 1990 to 2021

| Location | Deaths  (95% UI) | | DALYs  (95% UI) | |
| --- | --- | --- | --- | --- |
|  | 1990 | 2021 | 1990 | 2021 |
| Global | 2959777  (2067948,3852896) | 1127951  (748342,1508679) | 264203382  (184759129,343765298) | 100835580  (66876416,134894254) |
| Central Europe, Eastern Europe, and Central Asia | 64528  (47984,81090) | 13841  (9601,18108) | 5776582  (4294281,7260632) | 1059343  (723559,1397624) |
| High-income | 17553  (12677,22432) | 3554  (2724,4385) | 1571312  (1129415,2015717) | 403079  (292298,514257) |
| Latin America and Caribbean | 134640  (101029,168321) | 31168  (21827,40523) | 12054142  (9038946,15075987) | 2786413  (1946543,3627317) |
| North Africa and Middle East | 204372  (138520,270357) | 49671  (32527,66989) | 18272686  (12378564,24179173) | 4426346  (2891046,5979381) |
| South Asia | 1097522  (711940,1483447) | 356440  (222844,490852) | 97848828  (63601084,132127378) | 31911761  (19939985,43957835) |
| Southeast Asia, East Asia, and Oceania | 561444  (386891,737441) | 83422  (58264,108663) | 50183480  (34578459,65917755) | 7455551  (5198160,9720346) |
| Sub-Saharan Africa | 878836(594096,1164092) | 588524  (363979,813929) | 78417910  (53028330,103848322) | 46093787  (28049296,64215361) |
| Age |  |  |  |  |
| Early Neonatal | 641658  (456552,826785) | 381856  (245115,518667) | 57747301  (41088154,74408357) | 34366630  (22059647,46679804) |
| Late Neonatal | 453789  (348568,559050) | 164130  (127404,200888) | 40868576  (31391931,50348736) | 14783735  (11473199,18095408) |
| 1 to 5 months | 609855  (451160,768996) | 174321  (129998, 218845) | 55030174  (40719458, 69380889) | 15745996  (11746147, 19763760) |
| 6 to 11 months | 329948  (221823,438510) | 109460  (68436,150698) | 29683220  (19961044, 39444459) | 9855134  (6163633, 13565724) |
| 12 to 23 months | 330979  (217436,444645) | 90738  (54889,126742) | 29556800  (19418803,39705668) | 8109674  (4908600,11324841) |
| 2 to 4 years | 409493  (261523,557568) | 112377  (61042,163972) | 35967646  (22977990,48966699) | 9869560  (5372684,14390519) |
| 5 to 9 years | 119861  (71138,168656) | 53879  (34151,73704) | 10200180  (6032226,14377006) | 4670732  (2928543,6422418) |
| 10 to 14 years | 64194  (39749,88687) | 41188  (27307,55162) | 5149485  (3169522,7133485) | 3434119  (2223962,4651780) |

DALYs, disability-adjusted life-years; UI, uncertainty interval.

Table S2 Deaths (numbers and all-age rates) associated with and attributable to bacterial antimicrobial resistance, globally and by GBD super-region, 2021

| Location | Associate death counts  (95% UI) | | Associate death rate per 100000 (95% UI) | | Attributable death counts  (95% UI) | | Attributable death rate per 100000 (95% UI) | |
| --- | --- | --- | --- | --- | --- | --- | --- | --- |
|  | 1990 | 2021 | 1990 | 2021 | 1990 | 2021 | 1990 | 2021 |
| Global | 2443035  (1722153, 3164556) | 918652  (611600, 1225789) | 12.34  (8.69, 16.00) | 4.59  (3.06, 6.12) | 516742  (345795, 688340) | 209299  (136742, 282890) | 2.61  (1.74, 3.47) | 1.05  (0.69, 1.42) |
| Central Europe, Eastern Europe, and Central Asia | 52922  (39986, 65868) | 11191  (7882, 14501) | 5.02  (3.79, 6.26) | 1.45  (1.02, 1.88) | 11606  (7997, 15222) | 2650  (1719, 3607) | 1.10  (0.76, 1.45) | 0.34  (0.22, 0.47) |
| High-income | 14311  (10413, 18210) | 3656 (2688, 4626) | 0.74  (0.54, 0.94) | 0.22  (0.16, 0.28) | 3242  (2264, 4223) | 819  (602, 1036) | 0.17  (0.12, 0.22) | 0.05  (0.04, 0.06) |
| Latin America and Caribbean | 110906  (84187, 137679) | 25219  (17673, 32772) | 7.07  (5.36, 8.77) | 1.74  (1.22, 2.26) | 23733  (16842, 30643) | 5949  (4154, 7751) | 1.51  (1.07, 1.95) | 0.41  (0.29, 0.53) |
| North Africa and Middle East | 165869  (113191, 218655) | 39464  (25908, 53135) | 10.19  (6.95, 13.43) | 2.12  (1.39, 2.86) | 38503  (25329, 51703) | 10207  (6620, 13854) | 2.36  (1.56, 3.18) | 0.55  (0.36, 0.74) |
| South Asia | 905763  (590732, 1221038) | 287594  (179368, 396338) | 18.01  (11.71, 24.32) | 5.87  (3.67, 8.07) | 191758  (121207, 262409) | 68846  (43477, 94513) | 3.80  (2.39, 5.20) | 1.41  (0.90, 1.94) |
| Southeast Asia, East Asia, and Oceania | 464394  (323004, 606665) | 68011  (47536, 88501) | 8.44  (5.87, 11.03) | 1.79  (1.26, 2.33) | 97050  (63888, 130776) | 15411  (10728, 20162) | 1.76  (1.16, 2.38) | 0.41  (0.29, 0.53) |
| Sub-Saharan Africa | 728139  (497702, 958969) | 483182  (300669, 665766) | 24.07  (16.36, 31.79) | 8.70  (5.40, 11.99) | 150697  (96394, 205123) | 105342  (63310, 148164) | 4.96  (3.15, 6.77) | 1.89  (1.14, 2.67) |

GBD, Global Burden of Disease; UI, uncertainty interval.

Table S3 DALYs (numbers and all-age rates) associated with and attributable to bacterial antimicrobial resistance, globally and by GBD super-region, 2021

| Location | Associate DALY  (95% UI) | | Associate DALY rates  per 100000 (95% UI) | | Attributable DALY counts  (95% UI) | | Attributable DALY rates  per 100000 (95% UI) | |
| --- | --- | --- | --- | --- | --- | --- | --- | --- |
|  | 1990 | 2021 | 1990 | 2021 | 1990 | 2021 | 1990 | 2021 |
| Global | 217974818  (153776587, 282233039) | 82088442  (54616525, 109566120) | 1070.27  (754.21, 1386.62) | 398.86  (265.93, 531.83) | 46228564  (30982542, 61532259) | 18747138  (12259891, 25328134) | 226.65  (151.71, 301.87) | 91.35  (59.86, 123.28) |
| Central Europe, Eastern Europe, and Central Asia | 4736106  (3577054, 5896313) | 1000013  (703239, 1296803) | 437.46  (330.12, 544.92) | 126.29  (88.65, 163.94) | 1040476  (717227, 1364320) | 236709  (153628, 322269) | 96.09  (66.21, 126.02) | 29.89  (19.37, 40.72) |
| High-income | 1280962  (927147, 1637082) | 329368  (238546, 420530) | 64.25  (46.49, 82.12) | 19.11  (13.81, 24.43) | 290349  (202269, 378634) | 73711  (53752, 93727) | 14.56  (10.14, 18.99) | 4.27  (3.10, 5.44) |
| Latin America and Caribbean | 9926658  (7529007, 12329407) | 2254307  (1575394, 2933546) | 615.11  (466.54, 764.00) | 151.14  (105.54, 196.76) | 2127484  (1509939, 2746580) | 532106  (371149, 693772) | 131.77  (93.50, 170.13) | 35.68  (24.87, 46.54) |
| North Africa and Middle East | 14826594  (10111380, 19551902) | 3516807  (2302172, 4744110) | 885.25  (603.72, 1167.38) | 183.94  (120.32, 248.20) | 3446091  (2267184, 4627271) | 909539  (588874, 1235271) | 205.72  (135.33, 276.24) | 47.57  (30.77, 64.63) |
| South Asia | 80696044 (52729988, 108684262) | 25733590  (16034844, 35479643) | 1559.15  (1015.79, 2102.95) | 511.03  (319.68, 703.33) | 17152784  (10871096, 23443115) | 6178172  (3905142, 8478192) | 330.13  (208.58, 451.85) | 123.41  (78.26, 169.06) |
| Southeast Asia, East Asia, and Oceania | 41501142  (28859072, 54222788) | 6076183  (4238118, 7915623) | 733.32  (509.91, 958.14) | 155.98  (109.29, 202.69) | 8682338  (5719387, 11694967) | 1379368  (960042, 1804724) | 153.39  (101.03, 206.63) | 35.69  (24.93, 46.60) |
| Sub-Saharan Africa | 64942390  (44396651, 85518076) | 43148306  (26836168, 59466928) | 2083.62  (1416.03, 2752.18) | 754.90  (468.61, 1041.29) | 13475520  (8631679, 18330245) | 9430744  (5674967, 13257120) | 430.55  (274.00, 587.47) | 164.77  (98.94, 231.87) |

DALYs, disability-adjusted life-years; UI, uncertainty interval.

Table S4 The Top 15 Pathogens associated with sepsis burden in children globally in 2021

| Rank | Pathogens | Death Counts  (95% UI) | Pathogens | DALYs  (95% UI) |
| --- | --- | --- | --- | --- |
| 1 | Streptococcus pneumoniae | 141232  (104333，178132) | Klebsiella pneumoniae | 12491132  (9181109, 15801156) |
| 2 | Klebsiella pneumoniae | 140069  (103004，177133) | Streptococcus pneumoniae | 12421527  (9168363, 15674690) |
| 3 | Escherichia coli | 99105  (68031，130180) | Escherichia coli | 8957831  (6091422, 11824240) |
| 4 | Staphylococcus aureus | 81996  (59584，104407) | Staphylococcus aureus | 7306894  (5304259, 9309528) |
| 5 | Pseudomonas aeruginosa | 81654  (59860，103448) | Pseudomonas aeruginosa | 7266246  (5322943, 9209549) |
| 6 | Group B Streptococcus | 65561  (45387，85734) | Group B Streptococcus | 5880646  (4070836, 7690456) |
| 7 | Acinetobacter baumannii | 65267  (45190，85345) | Acinetobacter baumannii | 5812045  (4018172, 7605918) |
| 8 | Salmonella enterica serovar Typhi | 55506  (18259，92753) | Neisseria meningitidis | 4834820  (3098299, 6571342) |
| 9 | Neisseria meningitidis | 54435  (34943，73928) | Salmonella enterica serovar Typhi | 4717470  (1547865, 7887075) |
| 10 | Treponema pallidum | 51153  (8502，93804) | Treponema pallidum | 4613919  (773179, 8471006) |
| 11 | Candida spp. | 45489  (30892，60085) | Mycobacterium tuberculosis | 4263617  (3006496, 5551759) |
| 12 | Mycobacterium tuberculosis | 43870  (31389，56870) | Rotavirus | 4164607  (2133726, 6195489) |
| 13 | Non-typhoidal Salmonella | 41683  (15370，68211) | Candida spp. | 4062329  (2756462, 5368196) |
| 14 | Rotavirus | 40369  (21023，59716) | Non-typhoidal Salmonella | 3752547  (1313396, 6211201) |
| 15 | Other mycobacterium species | 38661  (21342，55980) | Other mycobacterium species | 3417267  (1870151, 4964384) |

DALYs, disability-adjusted life-years; UI, uncertainty interval.

Table S5 Global number of deaths, DALYs, and their corresponding ASR in children, by infectious syndrome, 2021

| Infectious Syndrome | Death counts | ASMR | DALYs | ASDR |
| --- | --- | --- | --- | --- |
|  | (95% UI) | per 100,000  (95% UI) | (95% UI) | per 100,000  (95% UI) |
| Bloodstream infections | 852484  (586901, 1118067) | 47.62  (32.77, 62.48) | 76314697  (52515486, 100113908) | 4265.82  (2933.89, 5597.75) |
| Lower respiratory infections | 593739  (466261, 721217) | 32.41  (25.47, 39.35) | 52962863  (41619154, 64306573) | 2895.18  (2276.98, 3513.39) |
| Diarrhea | 387370  (262542, 512198) | 21.08  (14.34, 27.81) | 37135804  (24926099, 49345508) | 2007.73  (1354.95, 2660.51) |
| Typhoid fever, paratyphoid fever, and invasive non-typhoidal Salmonella | 117416  (59187, 175646) | 6.18  (4.04, 8.32) | 10338815  (6763197, 13914434) | 552.92  (361.33, 744.50) |
| Meningitis | 115545  (75605, 155484) | 6.08  (3.06, 9.09) | 10065023  (5066236, 15063809) | 522.03  (262.43, 781.62) |
| Tuberculosis | 73454  (53571, 93856) | 4.07  (0.68, 7.46) | 6927474  (5003834, 8882136) | 367.56  (264.90, 471.96) |
| Sexually transmitted infections | 72489  (12063, 132914) | 3.90  (2.84, 4.99) | 6534416  (1096492, 11988687) | 366.74  (61.49, 672.78) |
| Peritoneal and intra-abdominal infections | 10808  (6970, 14646) | 0.56  (0.36, 0.76) | 931131  (592522, 1269740) | 48.47  (30.47, 66.47) |
| Urinary tract infections and pyelonephritis | 7709  (5448, 9969) | 0.41  (0.29, 0.53) | 684121  (483655, 884587) | 36.67  (20.19, 53.16) |
| Infections of the skin and subcutaneous systems | 6678  (3640, 9717) | 0.36  (0.19, 0.52) | 682884  (378472, 987295) | 36.10  (25.41, 46.79) |
| Endocarditis | 2200  (1475, 2926) | 0.12  (0.08, 0.15) | 195007  (130747, 259267) | 10.31  (6.88, 13.73) |
| Infections of bones, joints, and related organs | 247  (77, 451) | 0.01  (0.00, 0.02) | 45298  (13390, 80186) | 2.26  (0.67, 4.02) |

ASR, age-standardized rates; DALYs, disability-adjusted life-years; ASMR, age-standardized mortality rates; ASDR, age-standardized disability-adjusted life year; UI, uncertainty interval.

Table S6 Forecast of the number of deaths and death rates associated with and attributable to AMR among children, 2022–2050

| Years | Associated deaths  (95% UI) | Associated death rates  per 100,000 (95% UI) | Attributable deaths  (95% UI) | Attributable death rates  per 100,000 (95% UI) |
| --- | --- | --- | --- | --- |
| 2022 | 830657  (810733,851071) | 46.34  (45.38, 47.32) | 220969  (187278, 60721) | 10.58  (10.36, 10.82) |
| 2023 | 763009  (731977, 95356) | 43.27  (41.79, 44.81) | 215033  (180593, 256040) | 9.89  (9.55, 10.25) |
| 2024 | 694143  (649355, 42020) | 39.96  (37.77, 42.27) | 209256  (174226, 251328) | 9.13  (8.62, 9.68) |
| 2025 | 635235  (578691, 697303) | 37.18  (34.38, 40.20) | 203634  (168149, 246607) | 8.50  (7.85, 9.22) |
| 2026 | 579225  (511768, 55574) | 34.42  (31.02, 38.19) | 198163  (162340, 241892) | 7.87  (7.08, 8.76) |
| 2027 | 529327  (452634, 619013) | 31.97  (28.04, 36.45) | 192840  (156779, 237194) | 7.32  (6.40, 8.37) |
| 2028 | 483070  (398660, 85352) | 29.63  (25.23, 34.80) | 187659  (151449, 232525) | 6.78  (5.75, 8.00) |
| 2029 | 441223  (350664, 55168) | 27.50  (22.69, 33.33) | 182617  (146337, 227893) | 6.30  (5.17, 7.67) |
| 2030 | 402795  (307581, 7483) | 25.50  (20.35, 31.95) | 177711  (141428, 223303) | 5.84  (4.63, 7.36) |
| 2031 | 367829  (269332, 502346) | 23.66  (18.23, 30.70) | 172937  (136711, 218761) | 5.42  (4.15, 7.08) |
| 2032 | 335834  (235321, 479281) | 21.94  (16.30, 29.54) | 168291  (132177, 214273) | 5.03  (3.71, 6.82) |
| 2033 | 306658  (205244, 458184) | 20.36  (14.55, 28.47) | 163770  (127814, 209840) | 4.67  (3.31, 6.58) |
| 2034 | 279997  (178671, 438786) | 18.88  (12.97, 27.48) | 159370  (123615, 205467) | 4.33  (2.95, 6.36) |
| 2035 | 255665  (155274, 420961) | 17.52  (11.55, 26.57) | 155088  (119571, 201156) | 4.02  (2.62, 6.16) |
| 2036 | 233441  (134709, 404535) | 16.25  (10.26, 25.72) | 150922  (115676, 196908) | 3.73  (2.33, 5.97) |
| 2037 | 213152  (116679, 389391) | 15.07  (9.11, 24.93) | 146867  (111921, 192725) | 3.46  (2.07, 5.80) |
| 2038 | 194625  (100901, 375407) | 13.98  (8.08, 24.20) | 142922  (108302, 188609) | 3.21  (1.83, 5.63) |
| 2039 | 177709  (7123, 362485) | 12.97(7.15, 23.52) | 139082  (104811, 184560) | 2.98  (1.62, 5.48) |
| 2040 | 162263  (75113, 350532) | 12.03  (6.32, 22.89) | 135346  (101443, 180578) | 2.76  (1.43, 5.34) |
| 2041 | 148160  (64664, 339470) | 11.16  (5.58, 22.30) | 131710  (98194, 176665) | 2.56  (1.26, 5.21) |
| 2042 | 135282  (55589, 329226) | 10.35  (4.93, 21.75) | 128171  (95058, 172820) | 2.38  (1.11, 5.09) |
| 2043 | 123524  (47721, 319737) | 9.60  (4.34, 21.24) | 124728  (92030, 169043) | 2.21  (0.98, 4.98) |
| 2044 | 112788  (40911, 310944) | 8.91  (3.82, 20.77) | 121377  (89106, 165335) | 2.05  (0.86, 4.88) |
| 2045 | 102985  (35026, 302798) | 8.26  (3.36, 20.33) | 118116  (86282, 161696) | 1.90  (0.76, 4.78) |
| 2046 | 94034  (29949, 295250) | 7.66  (2.95, 19.92) | 114943  (83554, 158124) | 1.76  (0.66, 4.70) |
| 2047 | 85861  (25574, 288259) | 7.11  (2.59, 19.54) | 111855  (80918, 154619) | 1.64  (0.58, 4.61) |
| 2048 | 78398  (21811, 281788) | 6.59  (2.27, 19.18) | 108850  (78371, 151182) | 1.52  (0.51, 4.54) |
| 2049 | 71584  (18579, 275803) | 6.12  (1.98, 18.86) | 105926  (75909, 147811) | 1.41  (0.44, 4.47) |
| 2050 | 65362  (15807, 270272) | 5.67  (1.74, 18.55) | 103080  (73530, 144505) | 1.31  (0.39, 4.40) |

AMR, antimicrobial resistance; UI, uncertainty interval.

Table S7 Forecast of the number of DALYs and DALYs rates associated with and attributable to AMR among children, 2022–2050

| Years | Associated DALYs  **(95% UI)** | Associated DALYs Rate  **per 100,000 (95% UI)** | Attributable DALYs  **(95% UI)** | Attributable DALYs Rate  **per 100,000 (95% UI)** |
| --- | --- | --- | --- | --- |
| 2022 | 74215185  (72367985, 76109534) | 4144.19  (4056.15, 4234.15) | 6334749  (15950415, 16728343) | 948.64  (927.96, 969.78) |
| 2023 | 68184217  (65311501, 71183290) | 3871.20  (3735.22, 4012.13) | 4746669  (14180959, 15334946) | 887.06  (855.40, 919.89) |
| 2024 | 62026603  (57881704, 66468319) | 3575.36  (3374.84, 3787.79) | 2847117  (11916154, 13850813) | 819.01  (772.24, 868.60) |
| 2025 | 56769786  (51540394, 62529762) | 3327.29  (3071.03, 3604.94) | 1256571  (10053176, 12604015) | 762.82  (703.17, 827.53) |
| 2026 | 51764048  (45528601, 58853481) | 3080.76  (2769.42, 3427.10) | 9832354  (8344448, 11585569) | 706.22  (633.73, 786.99) |
| 2027 | 47308757  (40223750, 55641718) | 2862.19  (2502.27, 3273.88) | 8444018  (6751797, 10560365) | 656.54  (572.77, 752.54) |
| 2028 | 43175546  (35382206, 52685458) | 2653.11  (2249.79, 3128.73) | 7350665  (5493742, 9835240) | 608.61  (514.75, 719.57) |
| 2029 | 39437915  (31081986, 50040213) | 2463.03  (2022.48, 2999.53) | 6261601  (4325374, 9064566) | 565.29  (462.78, 690.50) |
| 2030 | 36004456  (27224578, 47615829) | 2284.24  (1812.53, 2878.72) | 5401416  (3427087, 8513145) | 524.34  (414.59, 663.14) |
| 2031 | 32880809  (23804173, 45418405) | 2119.88  (1622.81, 2769.21) | 4597733  (2650844, 7974498) | 486.81  (371.16, 638.50) |
| 2032 | 30022035  (20765806, 43404170) | 1966.45  (1449.71, 2667.37) | 3926012  (2043241, 7543686) | 451.68  (331.46, 615.50) |
| 2033 | 27415256  (18082467, 41564917) | 1824.67  (1293.45, 2574.08) | 3344714  (1557294, 7183685) | 419.27  (295.68, 594.52) |
| 2034 | 25032885  (15714810, 39876099) | 1692.78  (1151.96, 2487.49) | 2835479  (1172267, 6858452) | 389.07  (263.25, 575.02) |
| 2035 | 22858629  (13633179, 38326856) | 1570.63  (1024.58, 2407.69) | 2413499  (879519, 6622913) | 361.12  (234.08, 557.09) |
| 2036 | 20872608  (11806185, 36901485) | 1457.17  (909.88, 2333.64) | 2039456  (650023, 6398819) | 335.12  (207.80, 540.45) |
| 2037 | 19059482  (10206922, 35589951) | 1351.98  (806.94, 2265.14) | 1730950  (479402, 6249841) | 311.03  (184.24, 525.10) |
| 2038 | 17403662  (8809683, 34381201) | 1254.33  (714.65, 2201.57) | 1461936  (349239, 6119748) | 288.66  (163.10, 510.85) |
| 2039 | 15891803  (7591634, 33266805) | 1163.77  (632.10, 2142.64) | 1236539  (253213, 6038515) | 267.90  (144.21, 497.68) |
| 2040 | 14511218  (6531801, 32238499) | 1079.73  (558.36, 2087.94) | 1044628  (182139, 5991287) | 248.63  (127.34, 485.46) |
| 2041 | 13250605  (5611434, 31289422) | 1001.77  (492.61, 2037.20) | 881290  (130033, 5972868) | 230.75  (112.30, 474.15) |
| 2042 | 12099483  (4813621, 30413173) | 929.43  (434.07, 1990.10) | 744347  (92380, 5997557) | 214.15  (98.91, 463.67) |
| 2043 | 11048374  (4123284, 29604212) | 862.32  (382.03, 1946.42) | 627176  (65079, 6044161) | 198.75  (87.01, 453.98) |
| 2044 | 10088571  (3526963, 28857481) | 800.05  (335.84, 1905.91) | 529242  (45660, 6134458) | 184.45  (76.46, 445.01) |
| 2045 | 9212153  (3012718, 28168507) | 742.28  (294.90, 1868.37) | 445810  (31795, 6250890) | 171.19  (67.10, 436.72) |
| 2046 | 8411869  (2569968, 27533241) | 688.68  (258.66, 1833.62) | 375764  (22037, 6407453) | 158.88  (58.83, 429.07) |
| 2047 | 7681109  (2189375, 26948065) | 638.95  (226.62, 1801.48) | 316547  (15183, 6599784) | 147.45  (51.52, 422.03) |
| 2048 | 7013831  (1862717, 26409713) | 592.81  (198.34, 1771.81) | 266567  (10403, 6830426) | 136.84  (45.06, 415.55) |
| 2049 | 6404521  (1582770, 25915252) | 550.01  (173.41, 1744.47) | 224547  (7094, 7107137) | 127.00  (39.38, 409.61) |
| 2050 | 5848144  (1343207, 25462033) | 510.29  (151.45, 1719.34) | 189002  (4810, 7426020) | 117.87  (34.37, 404.18) |

DALYs, disability-adjusted life-years; UI, uncertainty interval.
